# Distinct Alpha-Fetoprotein Trajectories Preceding Hepatocellular Carcinoma Diagnosis: A Latent Class Mixed-Model Analysis

**DOI:** 10.3390/cancers18182900

**Published:** 2026-09-08

**Authors:** Apichat Kaewdech, Chanavee Toh, Pimsiri Sripongpun, Naichaya Chamroonkul, Pisit Tangkijvanich, Teerha Piratvisuth, Suthat Liangpunsakul, Thammasin Ingviya, Paramee Thongsuksai

**Affiliations:** 1 Gastroenterology and Hepatology Unit, Division of Internal Medicine, Faculty of Medicine, Prince of Songkla University, Hat Yai 90110, Songkhla, Thailand; apichat.ka@psu.ac.th (A.K.); spimsiri@medicine.psu.ac.th (P.S.); naichaya@gmail.com (N.C.); 2 Department of Clinical Research and Medical Data Science, Faculty of Medicine, Prince of Songkla University, Hat Yai 90110, Songkhla, Thailand; chanawee4@hotmail.com (C.T.); thammasin.i@psu.ac.th (T.I.); 3 Department of Biochemistry, Faculty of Medicine, Chulalongkorn University, Bangkok 10330, Thailand; pisittkvn@yahoo.com; 4 Center of Excellence in Hepatitis and Liver Cancer, Faculty of Medicine, Chulalongkorn University, Bangkok 10330, Thailand; 5 NKC Institute of Gastroenterology and Hepatology, Faculty of Medicine, Prince of Songkla University, Hat Yai 90110, Songkhla, Thailand; teerha.p@psu.ac.th; 6 Division of Gastroenterology and Hepatology, Department of Medicine, Indiana University School of Medicine, Indianapolis, IN 46202, USA; sliangpu@iu.edu; 7 Department of Biochemistry and Molecular Biology, Indiana University School of Medicine, Indianapolis, IN 46202, USA; 8 Department of Pathology, Faculty of Medicine, Prince of Songkla University, Hat Yai 90110, Songkhla, Thailand

**Keywords:** hepatocellular carcinoma, alpha-fetoprotein, latent class mixed model, trajectory, surveillance, biomarker

## Abstract

Background and Aims: Hepatocellular carcinoma (HCC) is frequently diagnosed at an advanced stage, and serum alpha-fetoprotein (AFP), the most widely used biomarker for HCC surveillance, exhibits substantial interpatient variability. However, longitudinal AFP patterns preceding HCC diagnosis and their clinical correlates remain incompletely characterized. We aimed to characterize pre-diagnostic AFP trajectories and identify clinical features associated with rising versus non-rising AFP patterns. Methods: We analyzed 529 patients with newly diagnosed HCC at Songklanagarind Hospital, each with at least three serum AFP measurements obtained within five years before diagnosis. Log-transformed AFP values were modeled using latent class mixed models with natural cubic splines and linear fixed and random effects. The optimal model was selected based on Bayesian Information Criterion (BIC), entropy, and clinical interpretability. Multinomial logistic regression and Cox proportional hazards models were used to evaluate associations between AFP trajectory class, baseline characteristics, and overall survival. Results: A two-class natural spline model (df = 3) provided the optimal fit (BIC = 9003). The non-rising trajectory (n = 440; 83.2%) showed stable AFP levels throughout follow-up, whereas the rising trajectory (n = 89; 16.8%) demonstrated exponential increase beginning 18–24 months before diagnosis. Compared to the non-rising group, patients in the rising group were significantly more likely to present with tumors >2 cm (78% vs. 62%; *p* = 0.009). The rising trajectory was associated with shorter overall survival in univariable analysis (hazard ratio [HR] 1.33, 95% confidence interval [CI] 1.02–1.73; *p* = 0.033), but not after adjustment for clinical and tumor characteristics, including Barcelona Clinic Liver Cancer stage (adjusted HR 1.01, 95% CI 0.77–1.33; *p* = 0.920). Conclusions: Two distinct AFP trajectories precede HCC diagnosis. Most patients did not demonstrate a substantial rise in AFP, underscoring the need for complementary surveillance biomarkers. Prospective validation in independent cohorts is required before AFP trajectory-based approaches can be incorporated into HCC surveillance practice. These findings are exploratory and hypothesis-generating rather than a validated clinical prediction tool.

## Simple Summary

Alpha-fetoprotein (AFP) is commonly used together with liver ultrasound to help detect hepatocellular carcinoma, the most common type of liver cancer. However, AFP levels vary considerably between patients, and a single measurement may not reflect changes occurring over time. We studied AFP measurements collected during the five years before liver cancer diagnosis to determine whether patients followed distinct patterns of change. Among 529 patients, approximately 83% had AFP levels that remained relatively stable, whereas 17% showed a rising pattern beginning approximately 18–24 months before diagnosis. Patients with a rising pattern were more likely to have larger tumors at diagnosis, although this pattern was not independently associated with survival after accounting for tumor stage. These findings highlight the limitations of relying on AFP alone and support further research into longitudinal AFP patterns together with other biomarkers and imaging methods.

## 1. Introduction

Hepatocellular carcinoma (HCC) is the sixth most commonly diagnosed cancer and the third leading cause of cancer-related death worldwide, with approximately 870,000 new cases and 760,000 deaths annually [1]. Despite advances in imaging, locoregional therapies, and systemic treatments, more than half of patients are diagnosed at intermediate or advanced stages at which curative treatment is no longer feasible [2,3]. Effective surveillance therefore remains a cornerstone of HCC management, particularly in patients with cirrhosis or chronic viral hepatitis [4].

Serum alpha-fetoprotein (AFP) is the longest-established biomarker in HCC surveillance and is recommended in combination with ultrasonography by several international guidelines [5,6,7]. Its diagnostic and prognostic performance, however, remains limited. Up to one-third of patients with early-stage HCC have AFP concentrations within the normal range, while non-malignant conditions such as active hepatitis or hepatic regeneration may produce spurious AFP elevations [8]. Because of this variability, single-time-point AFP thresholds capture only a fraction of the diagnostic information contained in serial AFP measurements.

Longitudinal modelling of AFP may complement cross-sectional cut-offs by characterizing patient-specific trends over time. Earlier studies have evaluated dynamic AFP measures, including delta AFP, AFP slope, AFP velocity, and algorithms incorporating serial AFP values, and have suggested that changes in AFP over time may provide additional information beyond its absolute concentration [9,10,11,12]. Persistently increasing AFP or a greater rate of AFP rise has been associated with a higher likelihood of HCC, while longitudinal approaches incorporating serial AFP measurements may improve discrimination compared with fixed AFP thresholds [13]. However, these approaches generally summarize AFP dynamics using predefined measures of change or model AFP as a continuous longitudinal process and may not fully capture heterogeneous, nonlinear patterns of AFP evolution among individual patients.

Whether discrete patterns of pre-diagnostic AFP behavior can be empirically distinguished, and whether such patterns correspond to distinct clinical phenotypes, remain incompletely understood. Latent class mixed models (LCMM) provide a principled framework for identifying unobserved subgroups of patients whose biomarker trajectories share a common shape, while accommodating the irregular sampling inherent in clinical practice [14]. Applying LCMM to serial pre-diagnostic AFP measurements may therefore provide a data-driven approach to characterize heterogeneity in AFP trajectories that is not captured by conventional measures such as AFP slope or velocity.

We hypothesized that the AFP trajectories observed over the five years preceding HCC diagnosis are not homogeneous across patients but cluster into a small number of clinically meaningful patterns. The aims of this study were (1) to identify and characterize latent AFP trajectories in a single-center cohort of patients with HCC, (2) to determine which baseline and tumor features are associated with trajectory class, and (3) to assess whether trajectory class carries independent prognostic information.

## 2. Methods

### 2.1. Study Design and Population

This was a retrospective cohort study of patients with newly diagnosed HCC who attended Songklanagarind Hospital, the tertiary referral center of Prince of Songkla University in southern Thailand, between January 2000 and December 2024. Eligible patients were adults (≥18 years) with HCC identified from the hospital tumor registry, as previously defined [15], and diagnosed in accordance with the American Association for the Study of Liver Diseases (AASLD) [5] and the European Association for the Study of the Liver (EASL) [6] guidelines, either by liver biopsy or by typical imaging features on contrast-enhanced computed tomography or magnetic resonance imaging. All eligible patients had at least three serum AFP measurements available within the five-year period preceding the date of HCC diagnosis. A minimum of three measurements was required to characterize longitudinal AFP patterns, as two measurements provide only a single interval change, whereas requiring four or more measurements could preferentially select patients undergoing more intensive surveillance. Patients were excluded if clinical data were incomplete.

The study was approved by the Human Research Ethics Unit (HREU) of the Faculty of Medicine, Prince of Songkla University (REC number 68-499-14-1) and was conducted in accordance with the principles of the 1975 Declaration of Helsinki. The requirement for written informed consent was waived given the retrospective nature of the analysis and the use of de-identified data.

### 2.2. Data Collection

Demographic data (age, sex, and body mass index), etiology of underlying liver disease (chronic hepatitis B, chronic hepatitis C, alcohol-related liver disease [ALD], metabolic dysfunction-associated steatotic liver disease [MASLD], autoimmune hepatitis, or other), antiviral treatment status, presence of cirrhosis and Child–Pugh score, relevant comorbidities (type 2 diabetes mellitus, dyslipidemia, and hepatic steatosis), and concomitant medications (statins, sodium-glucose cotransporter 2 [SGLT2] inhibitors, and glucagon-like peptide-1 [GLP-1] receptor agonists) were extracted from electronic medical records obtained from the Health Information System (HIS) of Songklanagarind Hospital. Tumor characteristics at diagnosis were recorded according to the Barcelona Clinic Liver Cancer (BCLC) staging system, including the largest tumor diameter, number of nodules, presence of macrovascular invasion, and presence and site of extrahepatic metastasis (bone, lung, adrenal gland, lymph node, and other). Vital status and date of death were ascertained from the hospital information system and the national death registry; follow-up was censored on 31 December 2025.

All serum AFP measurements obtained at the hospital during the five years before the HCC diagnosis date were retrieved. Serum AFP was measured using the ARCHITECT AFP assay (Abbott Laboratories). Measurements performed at outside hospitals were not included. AFP measurements were generally obtained at approximately 6-month intervals as part of routine HCC surveillance. All available measurements before HCC diagnosis, including those immediately preceding diagnosis, were retained because the objective of the study was to characterize the complete longitudinal evolution of AFP leading up to HCC diagnosis. Excluding measurements close to diagnosis could truncate the terminal portion of the trajectory and obscure a pre-diagnostic rise in AFP. AFP values were log-transformed (natural logarithm) prior to analysis to stabilize variance and accommodate the right-skewed distribution of the data.

### 2.3. Statistical Analysis

Baseline characteristics were summarized as medians with first and third quartiles for continuous variables and as counts with percentages for categorical variables. Between-class comparisons used the Wilcoxon rank-sum test for continuous variables and Pearson’s chi-squared or Fisher’s exact test for categorical variables.

Latent class mixed models were fitted using the lcmm package (version 2.1) in R (version 4.4) to identify distinct trajectories of log-AFP as a function of time before HCC diagnosis. Four functional forms for the time variable were evaluated: a linear specification and natural cubic splines with three, four, and five degrees of freedom. Random intercept and random slope terms were included for each patient. Models with two through five latent classes were estimated for each functional form, initialized from the one-class solution. The optimal number of classes and functional form of time were selected jointly based on the Bayesian Information Criterion (BIC), entropy (a measure of classification certainty, with values approaching 1 indicating better class separation), the proportion of patients assigned to the smallest class, and the clinical interpretability of the resulting trajectories.

Following class assignment using posterior class-membership probabilities, factors associated with class membership were examined using multinomial logistic regression, with age, sex, cirrhosis status, etiology (viral versus non-viral), largest tumor diameter, tumor number, and five-year era of diagnosis included as covariates. Overall survival was estimated by the Kaplan–Meier method and compared across classes using the log-rank test. The association between trajectory class and overall survival was further evaluated using Cox proportional-hazards regression, adjusted for age, sex, largest tumor diameter, and BCLC stage (0, A, or B–D). All statistical tests were two-sided, and a *p*-value of less than 0.05 was considered statistically significant.

## 3. Results

### 3.1. Patient Characteristics

A total of 7355 patients with HCC registered at Songklanagarind Hospital between 2000 and 2024 were screened for eligibility (Supplementary Table S1). Of these, 530 met the inclusion criteria of at least three serum AFP measurements within the five years preceding HCC diagnosis; one patient was subsequently excluded because of incomplete data. The final cohort comprised 529 patients (Supplementary Figure S1). The median age at HCC diagnosis was 63 years (interquartile range [IQR], 56–70), and 351 patients (66%) were men. Chronic viral hepatitis (hepatitis B or C) was the predominant underlying liver disease, present in 369 patients (70%), and cirrhosis was present at the time of HCC diagnosis in 517 patients (98%). The largest tumor exceeded 2 cm in diameter in 341 patients (64%), and 171 (32%) had multifocal disease. The distribution of underlying liver disease etiology was similar between the AFP trajectory classes: HBV accounted for 45% and 46%, HCV for 25% and 24%, and other etiologies for 30% and 30% of patients in the non-rising and rising classes, respectively (SMD = 0.03, *p* = 0.984). In contrast, patients in the rising class were more likely to have their largest HCC nodule >2 cm than those in the non-rising class (78% vs. 62%; SMD = 0.34, *p* = 0.005). Patients had a median of 7 AFP measurements (IQR, 4–10) during the five years preceding HCC diagnosis, with a median interval between consecutive measurements of 164 days (IQR, 107–197). The number of AFP measurements and measurement intervals were similar between the non-rising and rising trajectory classes (6 vs. 7 measurements, *p* = 0.753; and 161 vs. 175 days, *p* = 0.124, respectively). Detailed baseline characteristics stratified by trajectory class are presented in Table 1.

### 3.2. Observed AFP Trajectories

**Figure 1:**
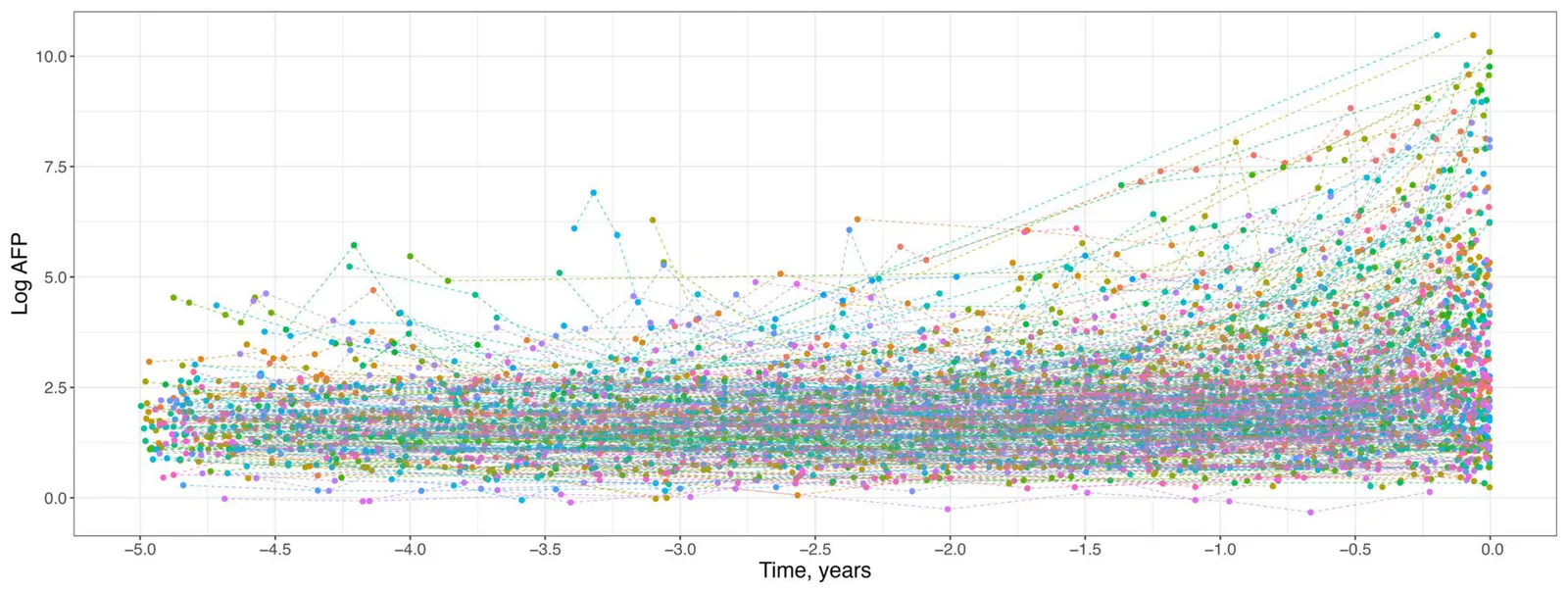
Spaghetti plot of all available serum AFP measurements during the five years preceding HCC diagnosis (n = 529 patients). Each thin colored line represents one patient. The y-axis shows AFP on the natural-logarithmic scale; the x-axis shows the number of days before HCC diagnosis (time 0). The wide spread of trajectories motivated the use of latent class mixed models.

The pre-diagnostic serum AFP trajectories of all 529 patients are displayed in Figure 1. AFP values exhibited substantial interindividual variability throughout the five-year observation window: the majority of patients showed stable concentrations within the conventional normal range, whereas a sizeable minority demonstrated a marked exponential rise during the final months before diagnosis. This heterogeneity motivated the application of an unsupervised latent-class approach to identify discrete underlying trajectory patterns.

### 3.3. Selection of the Latent Class Model

**Figure 2:**
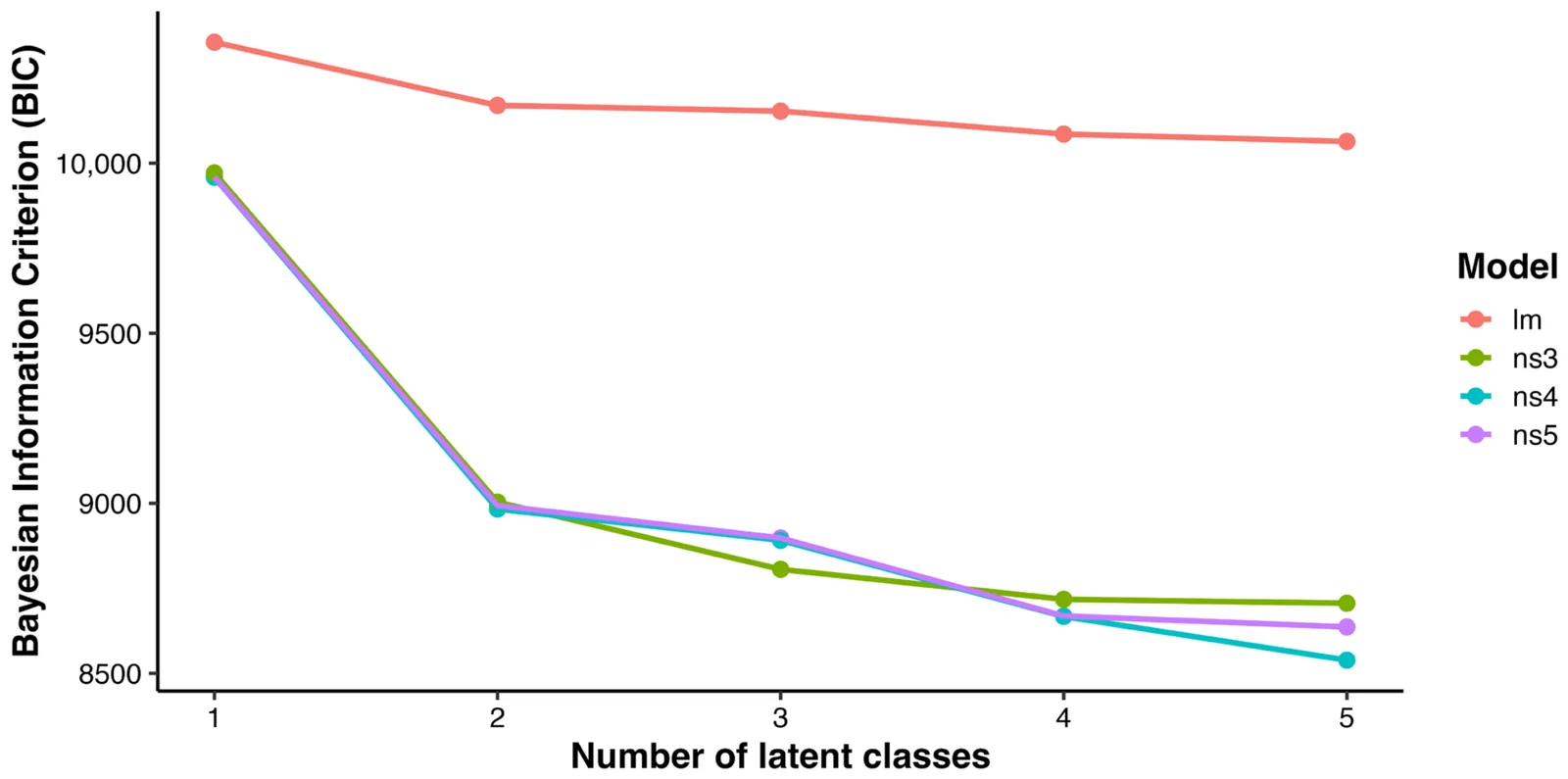
Model selection: Bayesian Information Criterion (BIC) for one- to five-class LCMM solutions across four functional forms for time. The largest drop in BIC was observed between the one-class and two-class solutions for all spline specifications. Linear specifications (red) were inferior at every class count. Additional classes beyond two yielded only marginal BIC gains and produced very small subgroups; the two-class natural-spline solution with three degrees of freedom was retained.

The BIC declined sharply from the one-class to the two-class natural-spline solution (9971 to 9003) but improved only marginally with additional classes (Figure 2 and Table 2). Models with three or more classes yielded small subgroups (2.2–4.0% of patients) that were not clinically distinguishable from the principal patterns and were therefore considered to offer limited additional value. The two-class natural-spline model with three degrees of freedom (entropy, 0.88) was selected as the primary model on the grounds of parsimony, classification certainty, and interpretability.

### 3.4. Class-Specific AFP Trajectories

**Figure 3:**
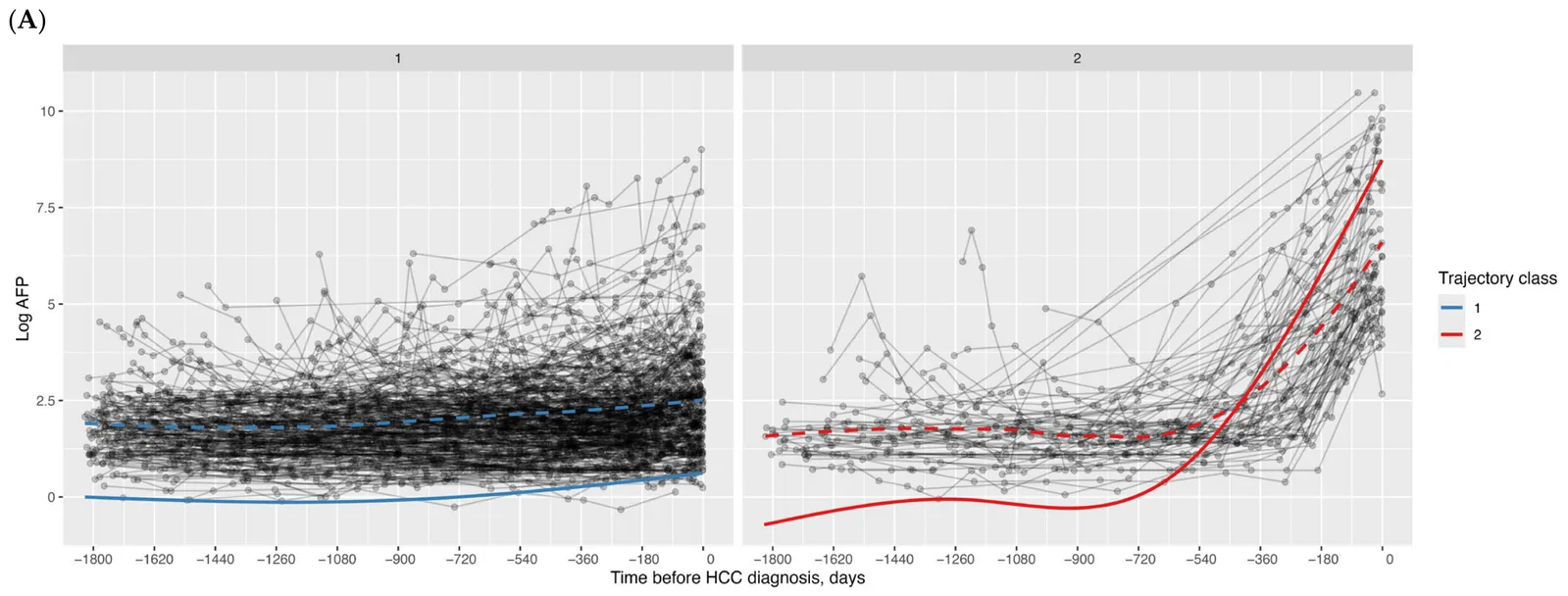

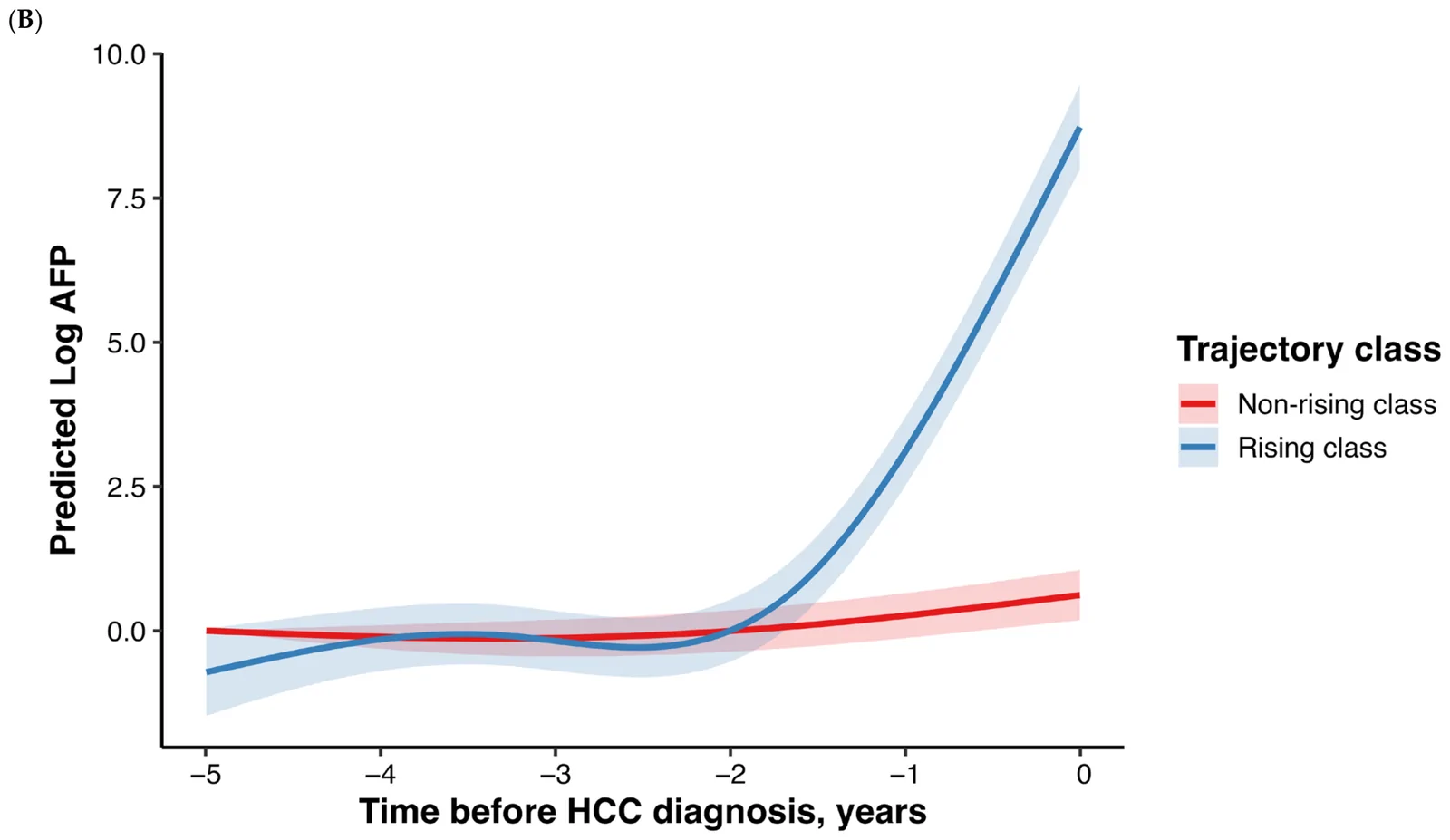
Class-specific AFP trajectories from the two-class natural-spline (df = 3) latent class mixed model. (**A**) Observed individual trajectories by class. Left panel: non-rising class (n = 440); right panel: rising class (n = 89). Each grey line represents an individual patient, and the solid lines represent the model-predicted AFP trajectories derived from the latent class mixed model (LCMM), whereas the dashed lines represent LOESS-smoothed trajectories estimated directly from the observed data. The y-axis is AFP on the natural-logarithmic scale; the x-axis is time before HCC diagnosis (days). (**B**) Model-predicted class-mean trajectories. The two curves are the marginal class-mean predictions from the latent class mixed model on the natural-logarithmic AFP scale, plotted against time before HCC diagnosis (years). The non-rising class (Class 1, red curve) remains essentially flat throughout the five-year window, whereas the rising class (Class 2, teal curve) shows a steep exponential surge during the final 18–24 months.

The two-class model identified one large class (Class 1: 440 patients, 83.2%) characterized by an essentially stable AFP trajectory throughout the five-year observation window, hereafter referred to as the “non-rising” class, and a smaller class (Class 2: 89 patients, 16.8%) characterized by an exponential surge in AFP that became apparent approximately 18–24 months before HCC diagnosis, hereafter referred to as the “rising” class (Figure 3A). The model-predicted class-mean trajectories (Figure 3B) confirmed a near-flat curve for the non-rising class throughout the observation window, whereas the rising class showed a gradual decline from five to approximately two years before diagnosis, followed by an exponential increase thereafter. This inflection reflects the model-predicted class-mean spline trajectory rather than an observed change point in any individual patient; because AFP measurements were obtained at irregular, patient-specific intervals, the timing of AFP acceleration in an individual patient may differ from this population-average estimate.

### 3.5. Predictors of Class Membership

**Figure 4:**
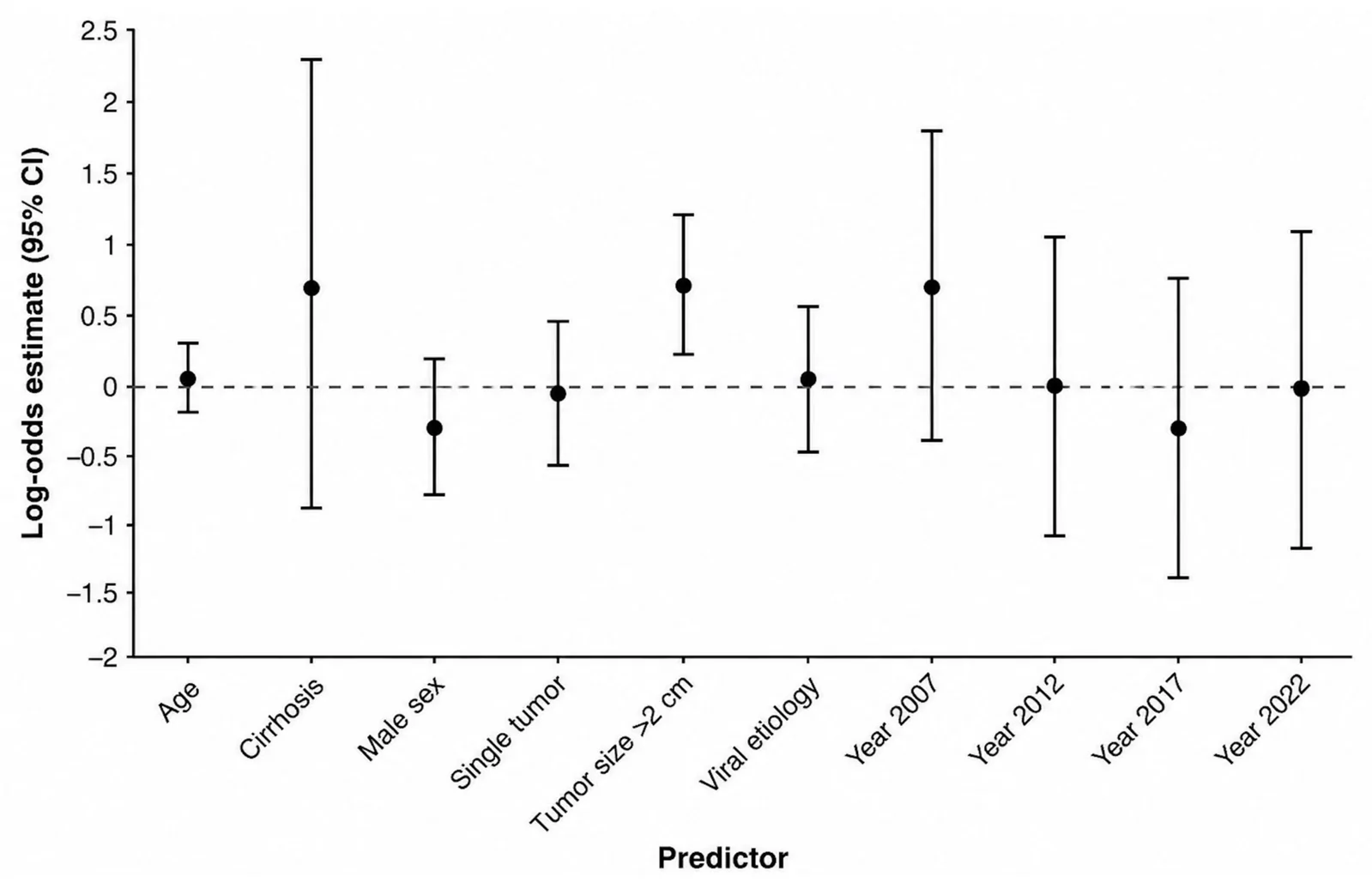
Forest plot of the multinomial logistic regression model for trajectory-class membership (rising versus non-rising). Points indicate log-odds estimates and whiskers indicate 95% confidence intervals. A largest tumor diameter >2 cm and the era of HCC diagnosis differed significantly between classes; age, sex, cirrhosis, viral etiology, and tumor multiplicity did not.

In the multinomial logistic regression model (Figure 4), the largest tumor diameter was significantly associated with class membership (largest tumor > 2 cm: likelihood-ratio χ^2^ = 6.85, *p* = 0.009). Patients in the rising class were more likely than those in the non-rising class to have tumors exceeding 2 cm at diagnosis (78% vs. 62%). Age, sex, presence of cirrhosis, underlying etiology, and tumor number did not differ significantly between classes after adjustment.

### 3.6. Survival Outcomes

During follow-up, 377 of 529 patients (71.3%) died, including 309 deaths in the non-rising AFP trajectory class and 68 deaths in the rising AFP trajectory class.

In univariable analysis, patients in the rising AFP trajectory class had a higher risk of mortality than those in the non-rising class (HR 1.33, 95% CI 1.02–1.73, *p* = 0.033). However, after adjustment for age, sex, cirrhosis, tumour size, and BCLC stage, AFP trajectory class was no longer independently associated with mortality (adjusted HR 1.01, 95% CI 0.77–1.33, *p* = 0.920).

In the multivariable Cox proportional hazards model, cirrhosis (adjusted HR 5.25, 95% CI 1.67–16.45, *p* = 0.004), larger tumour size (adjusted HR 1.17 per 1-cm increase, 95% CI 1.12–1.22, *p* < 0.001), and BCLC stage B–D (adjusted HR 2.11, 95% CI 1.56–2.86, *p* < 0.001) were independently associated with increased mortality. In contrast, age, sex, AFP trajectory class, and BCLC stage A were not significantly associated with overall survival after adjustment (Table 3).

To assess whether multicollinearity could explain the attenuation of the AFP trajectory class effect in the multivariable model, we calculated the generalised variance inflation factor (GVIF) for each predictor. All GVIF(^1/(2×Df)^) values were below 1.20, well within the conventional threshold of 2.5, confirming the absence of meaningful collinearity (Supplementary Table S2).

### 3.7. Subgroup Analysis by Tumor Size at Diagnosis

To explore whether the association between AFP trajectory class and clinical outcomes differed according to tumor burden at diagnosis, we conducted exploratory subgroup analyses stratifying the cohort by HCC size: patients with tumor diameter >2 cm (n = 302) and patients with tumor diameter ≤2 cm (n = 227). Within each subgroup, baseline characteristics were compared between AFP trajectory classes (Non-rising vs. Rising) and overall survival was assessed using multivariable Cox proportional-hazards regression adjusting for age, sex, AFP trajectory class, tumor size, and HCC stage.

Subgroup 1: Tumor > 2 cm (n = 302). Among patients with tumors >2 cm, the Rising class (n = 62) had significantly higher median Log AFP values compared with the Non-rising class (n = 240) (5.71 vs. 2.08, *p* < 0.001) and larger tumor diameter at diagnosis (4.65 vs. 3.40 cm, *p* = 0.002). In multivariable Cox regression (Supplementary Table S3), AFP trajectory class was not independently associated with overall survival within this subgroup (adjusted HR = 1.27, 95% CI 0.93–1.73; *p* = 0.14). Significant predictors of survival were tumor size (adjusted HR = 1.18 per cm increase, 95% CI 1.13–1.24; *p* < 0.001) and intermediate to advanced HCC stage (others vs. BCLC-0-A: adjusted HR = 2.22, 95% CI 1.71–2.89; *p* < 0.001).

Subgroup 2: Tumor ≤ 2 cm (n = 227). Among patients with tumors ≤2 cm, the Rising class (n = 27) again showed markedly elevated Log AFP levels compared with the Non-rising class (n = 200) (5.84 vs. 2.05, *p* < 0.001), whereas other clinical characteristics including tumor diameter, tumor number, liver function, and comorbidities did not differ significantly between classes. In multivariable Cox regression (Supplementary Table S4), AFP trajectory class was not significantly associated with overall survival (adjusted HR = 1.11, 95% CI 0.66–1.88; *p* = 0.7). HCC stage was the only significant predictor of survival in this subgroup (BCLC-A vs. 0: adjusted HR = 1.59, 95% CI 1.08–2.33; *p* = 0.019).

### 3.8. Sensitivity Analysis

As a sensitivity analysis, LCMM was refitted using raw (untransformed) AFP values. The two-class solution remained the best-fitting model by BIC; however, the Rising class comprised fewer than 5% of patients, a proportion too small to yield stable parameter estimates or clinically interpretable class trajectories. The natural-logarithm transformation normalises the skewed AFP distribution, stabilises variance, and is consistent with clinical practice in which AFP is routinely interpreted on a logarithmic scale. The two-class solution using natural-logarithm (ln)-AFP was therefore retained as the primary analysis, consistent with the transformation described in Section 2.2.

## 4. Discussion

In this single-center cohort of 529 patients with HCC, we identified two empirically distinct trajectories of serum AFP during the five years preceding HCC diagnosis. The majority of patients (≈83%) showed a non-rising trajectory, in which AFP remained stable and near the upper limit of normal even as malignant transformation progressed. The remaining patients (≈17%) followed a rising trajectory characterized by an exponential surge beginning approximately 18 to 24 months before diagnosis. These two patterns differed not only in shape but also in the AFP level at diagnosis and in tumor burden at presentation.

**Figure 5:**
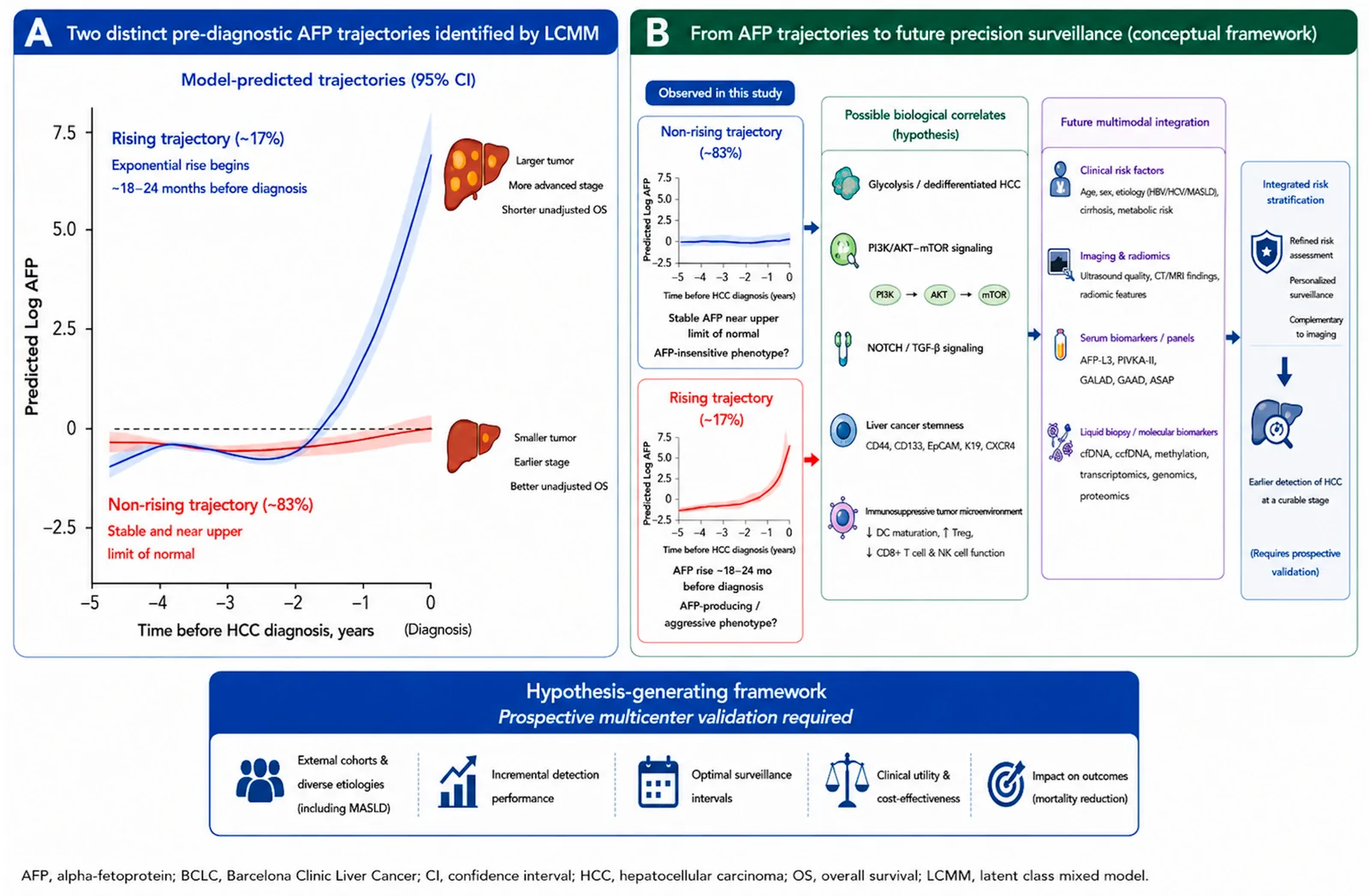
Pre-diagnostic AFP trajectory patterns and a conceptual framework for future multimodal HCC surveillance.

The clinical implications of these findings are several. Identifying latent AFP trajectories may provide information that is not captured by conventional AFP thresholds because threshold-based approaches classify patients according to a single AFP value and may therefore overlook meaningful changes occurring below the diagnostic cutoff. In contrast, trajectory-based analysis incorporates serial measurements and the pattern of change over time, allowing identification of a subgroup with a progressive AFP rise even before marked AFP elevation occurs. Importantly, our study was not designed to demonstrate that trajectory-based surveillance improves diagnostic performance compared with conventional AFP thresholds, and prospective studies including patients with and without HCC are required to establish its incremental value (Figure 5).

First, the dominant non-rising pattern provides empirical support for the well-recognized insensitivity of AFP-based surveillance in a large proportion of patients. Although guidelines continue to endorse AFP in combination with ultrasonography [3,5,6,7], additional biomarkers may be needed to improve early detection, particularly in patients whose tumors do not produce a measurable AFP signal. Candidate markers include Protein Induced by Vitamin K Absence or Antagonist-II (PIVKA-II; des-γ-carboxy prothrombin), AFP-L3, composite biomarker models such as the GALAD score (incorporating age, sex, AFP, AFP-L3, and PIVKA-II), the GAAD and ASAP scores, which do not require AFP-L3, and emerging cell-free DNA–based assays [16,17,18].

Second, the rising trajectory, although present in a minority, was associated with more advanced disease at presentation and shorter unadjusted survival. This finding suggests that a progressive increase in serial AFP may contain clinically relevant information beyond that provided by a single AFP threshold and warrants prospective evaluation as a potential trigger for additional imaging or intensified surveillance. This finding is supported by a recent large study demonstrating that a lower AFP cutoff of ≥10 ng/mL provided higher sensitivity for HCC detection than the traditional cutoff of ≥20 ng/mL (41.9% vs. 31.7%, respectively) [19].

Third, the trajectory effect on overall survival was largely mediated by tumor stage, consistent with the hypothesis that an AFP surge reflects, rather than independently drives, more aggressive tumor biology [20]. In subgroup analyses stratified by tumor size, AFP levels at HCC diagnosis were significantly higher in the rising than in the non-rising trajectory class among patients with both tumors ≤2 cm and >2 cm. However, a significant difference in tumor diameter between trajectory classes was observed only among patients with tumors >2 cm. This subgroup finding should be distinguished from the multivariable analysis of predictors of trajectory-class membership, in which a largest tumor diameter >2 cm was significantly associated with membership in the rising AFP class. Notably, AFP trajectory class was not an independent predictor of overall survival in either subgroup after adjustment for tumor characteristics, suggesting that its prognostic relevance is mediated through its association with tumor size at presentation. These findings are consistent with the hypothesis that a rising AFP trajectory reflects accelerating tumor growth, leading to larger, more advanced tumors at the time of clinical detection. Collectively, these observations provide a rationale for prospective studies evaluating whether incorporation of AFP trajectories into surveillance algorithms can improve early HCC detection beyond conventional AFP thresholds.

The biological basis for a rising, exponential pre-diagnostic AFP trajectory may reflect the emergence of a biologically distinct, AFP-producing tumor phenotype rather than simply increasing tumor burden. Recent molecular classification of HCC demonstrated that the glycolysis subclass had substantially higher serum AFP levels than the rich-metabolism subclass (median 235 vs. 7 ng/mL, *p* < 0.001) and was characterized by larger tumors, more frequent vascular invasion, poorer differentiation, and predominant correspondence to the prognostically unfavorable Hoshida S1/S2 subclasses. Within the glycolysis subclass, distinct PI3K/mTOR-High and NOTCH/TGF-β-High molecular phenotypes were identified [21]. AFP itself may also contribute to aggressive tumor biology rather than merely serving as a circulating marker. Experimental evidence suggests that AFP promotes liver cancer stem-cell expansion through PI3K/AKT signaling and increases the expression of stem-cell markers, including CD133, CD44, EpCAM, K19, and CXCR4. AFP may also promote an immunosuppressive tumor microenvironment by impairing dendritic-cell maturation, promoting regulatory T-cell differentiation, and suppressing CD8+ T-cell and natural killer cell function [20]. If the rising trajectory in our cohort reflects emergence or clonal expansion of such an AFP-producing, dedifferentiated subpopulation, this would be consistent with its association with larger tumor size and shorter unadjusted survival. However, because histological grade, TP53 mutation status, and molecular subclass were not available in this retrospective cohort, this mechanistic interpretation remains speculative and warrants direct testing in studies that pair longitudinal AFP trajectories with tumor molecular profiling.

With the rising incidence of non-viral HCC, particularly MASLD-related HCC, evaluating the diagnostic performance of AFP in this population has become increasingly important. In our cohort, etiology-specific analysis showed no significant association between underlying liver disease and AFP trajectory class membership. HBV accounted for 45% and 46% of patients in the non-rising and rising classes, respectively, HCV for 25% and 24%, and other etiologies for 30% in both classes (SMD = 0.03, *p* = 0.984). Previous studies have reported that MASLD-related HCC is less likely to demonstrate marked AFP elevation compared with viral-related HCC, with lower proportions of patients exhibiting AFP ≥ 20 ng/mL and generally lower AFP levels at diagnosis [22,23]. In contrast to those cross-sectional observations, our longitudinal trajectory analysis showed that AFP trajectory class membership (non-rising versus rising) did not differ significantly according to underlying etiology (viral versus non-viral). These findings suggest that, although absolute AFP levels at the time of HCC diagnosis may vary across disease etiologies, the temporal dynamics of AFP change preceding diagnosis may be broadly similar regardless of the underlying etiology. This observation further supports the value of longitudinal AFP monitoring over reliance on a single AFP threshold.

Our findings extend prior cross-sectional analyses of AFP performance by quantifying the heterogeneity of pre-diagnostic AFP trajectories [8]. Two-class structures resembling ours have been reported in cohort studies of patients with cirrhosis using joint longitudinal–survival models [11,24,25], and the observation that approximately one in six patients with HCC exhibits a steep terminal rise is consistent with the proportion of patients in whom delta-AFP–based scores outperform single-threshold approaches [13,26,27]. The exponential pattern observed in the rising trajectory class, beginning approximately two years before HCC diagnosis, may reflect underlying tumor growth kinetics and is broadly consistent with previously reported tumor doubling-time estimates derived from serial imaging studies [28].

Several alternative approaches can be used to analyze longitudinal biomarker data. Joint longitudinal–survival models are particularly useful when the primary objective is to simultaneously model longitudinal biomarker changes and time-to-event outcomes, whereas generalized additive mixed models can flexibly characterize nonlinear population-level and patient-specific trajectories without identifying discrete latent subgroups [29]. Growth mixture models, similar to LCMM, can identify latent subgroups with distinct longitudinal patterns but differ in their parameterization and modeling assumptions [30]. We selected LCMM because our primary objective was to empirically identify unobserved subgroups of patients with distinct nonlinear pre-diagnostic AFP trajectories while accounting for repeated and irregularly timed measurements. These alternative approaches may provide complementary information and warrant consideration in future validation studies.

Strengths of the study include the relatively large number of patients with serial pre-diagnostic AFP measurements, the long observation window of up to five years, the use of a principled latent-class framework that does not impose a fixed trajectory shape, and the explicit comparison across multiple functional forms for time, which strengthens confidence in the two-class solution.

Several limitations of this study warrant consideration. First, the study population was drawn from a single tertiary referral center in southern Thailand, and only 529 of 7355 screened patients (7.2%) had sufficient serial AFP measurements for trajectory analysis. Comparison with excluded patients demonstrated differences in age, sex, AFP measurement frequency, and several laboratory characteristics, indicating potential selection bias. Patients with repeated pre-diagnostic AFP measurements may have had greater healthcare engagement or more consistent surveillance than the broader HCC population. Although AFP measurements were generally obtained at approximately 6-month intervals as part of routine HCC surveillance, detailed information regarding enrollment in structured surveillance programs and surveillance adherence was not consistently available throughout the study period. In addition, chronic viral hepatitis remained the predominant etiology of HCC in our cohort. This case mix may not be representative of Western or other Asian cohorts in which MASLD- or ALD-related HCC accounts for a larger proportion of cases, and the relative size of the rising class may therefore differ in such populations. Accordingly, the identified trajectories may not be fully generalizable to the broader HCC population, particularly patients without regular surveillance or longitudinal biomarker testing, and should be externally validated in geographically and etiologically diverse cohorts. Second, the retrospective design and reliance on routine clinical practice resulted in heterogeneous AFP sampling intervals across patients, with some individuals having measurements concentrated in the period immediately preceding diagnosis and relatively sparse data in the earlier portions of the five-year window. AFP measurements immediately preceding HCC diagnosis were intentionally retained because the objective of this study was to characterize the complete pre-diagnostic AFP trajectory, including a potential terminal rise. Excluding these measurements could truncate the trajectory and obscure the pattern of interest. Nevertheless, measurements obtained close to diagnosis may have had greater influence on trajectory classification and, in some patients, may have reflected intensified clinical evaluation around the time of HCC detection. Although LCMM accommodates irregular sampling through patient-specific random effects, trajectory estimates during the period more than four years before diagnosis rest on comparatively few observations per patient, and the shape of early AFP dynamics should be interpreted with appropriate caution. Third, several potentially important time-varying factors that may influence AFP could not be fully incorporated because of incomplete longitudinal ascertainment. These included on-treatment viral load and virological response, hepatic inflammatory activity and liver injury at the time of individual AFP measurements, and detailed surveillance intensity. Approximately half of the cohort was receiving antiviral therapy, with no significant difference in antiviral treatment status between trajectory classes. Available liver biochemical parameters also did not suggest substantial biochemical hepatitis. Furthermore, AFP sampling intensity was broadly comparable between classes: the median numbers of AFP measurements were 6 and 7 (*p* = 0.753), and the median intervals between measurements were 161 and 175 days (*p* = 0.124) in the non-rising and rising classes, respectively. These findings reduce the likelihood that the observed trajectory differences were primarily attributable to antiviral treatment status, active hepatitis, or differential AFP sampling frequency. Nevertheless, transient hepatic inflammation or liver injury and changes in viral activity or treatment response could alter AFP independently of tumor progression, while differences in surveillance imaging intensity could also influence the timing of HCC detection. Residual confounding from these time-varying factors therefore cannot be completely excluded. Fourth, class assignment in LCMM is probabilistic; patients are allocated to the class with the highest posterior membership probability but carry residual uncertainty, particularly those near the class boundary. Although the entropy of the selected model was high (0.88), misclassification of borderline patients could attenuate observed associations with clinical outcomes. Fifth, because AFP values below the assay detection limit were not uniformly available, left-censoring of low AFP values may introduce a degree of informative missingness that the current analysis does not fully address. Finally, although we adjusted for the principal confounders available in the dataset, residual confounding by unmeasured factors, including specific antiviral regimens, medication adherence, alcohol use intensity, and the presence of hepatic inflammation at the time of each AFP measurement, cannot be excluded.

## 5. Conclusions

Two distinct patterns of AFP behavior can be discerned in the five years before HCC diagnosis: a dominant non-rising trajectory in approximately 83% of patients and a rising trajectory in approximately 17%, in whom AFP increases exponentially from roughly 18–24 months before diagnosis. Recognition of the non-rising pattern underscores the limitations of AFP-only surveillance and reinforces the need for complementary biomarkers, whereas detection of a rising pattern may warrant further prospective evaluation as a trigger for intensified surveillance. Prospective validation of trajectory-based risk stratification in independent cohorts and integration with novel biomarkers represent logical next steps. These trajectory classes represent exploratory, hypothesis-generating findings derived from a single retrospective cohort and should not yet be regarded as a validated clinical prediction tool for HCC surveillance.

## Figures and Tables

**Table 1:** Baseline demographic, clinical, and tumor characteristics of the study cohort stratified by AFP trajectory class.

Characteristic	Overall (N = 529)	Non-Rising Class (n = 440)	Rising Class (n = 89)	SMD	95% CI	*p*-Value
AFP at diagnosis, log-ng/mL (median, IQR)	2.40 (1.53, 3.87)	2.08 (1.38, 2.77)	5.71 (4.72, 7.39)	−2.3	−2.6, −2.1	<0.001
Age at HCC diagnosis, years (median, IQR)	63 (56, 70)	62 (56, 70)	63 (58, 69)	−0.04	−0.27, 0.18	0.455
BMI, kg/m^2^ (median, IQR)	24.8 (22.7, 27.7)	24.8 (22.7, 27.9)	24.2 (22.2, 27.4)	0.16	−0.07, 0.38	0.176
Sex, n (%)				0.09	−0.14, 0.31	0.5
Female	178 (34%)	145 (33%)	33 (37%)			
Male	351 (66%)	295 (67%)	56 (63%)			
Etiology, n (%)				0.03	−0.19, 0.26	0.984
HBV	238 (45%)	197 (45%)	41 (46%)			
HCV	131 (25%)	110 (25%)	21 (24%)			
Others	160 (30%)	133 (30%)	27 (30%)			
Cirrhosis, n (%)	517 (98%)	429 (98%)	88 (99%)	0.10	0.12, 0.33	0.701
Child-Pugh Class, n (%)				0.17	−0.06, 0.40	0.332
A	419 (79.2%)	353 (80%)	66 (74%)			
B	95 (18%)	74 (17%)	21 (24%)			
C	15 (2.8%)	13 (3.0%)	2 (2%)			
Antiviral treatment, n (%)				0.06	−0.16, 0.29	0.894
None	245 (46%)	206 (47%)	39 (44%)			
HBV antiviral	197 (37%)	162 (37%)	35 (39%)			
HCV antiviral	82 (16%)	68 (15%)	14 (16%)			
Both HCV & HBV	5 (0.9%)	4 (0.9%)	1 (1.1%)			
Type 2 Diabetes Mellitus, n (%)	190 (36%)	159 (36%)	31 (35%)	0.03	−0.20, 0.26	0.815
Dyslipidemia, n (%)	144 (27%)	118 (27%)	26 (29%)	0.05	−0.17, 0.28	0.643
Fatty liver disease, n (%)	23 (4.3%)	17 (3.9%)	6 (6.7%)	0.13	−0.10, 0.36	0.251
Statins, n (%)	95 (18%)	76 (17%)	19 (21%)	0.10	−0.12, 0.33	0.361
SGLT2 inhibitors, n (%)	4 (0.8%)	3 (0.7%)	1 (1.1%)	0.05	−0.18, 0.27	0.522
GLP-1 receptor agonist, n (%)	3 (0.6%)	3 (0.7%)	0 (0%)	0.12	−0.11, 0.35	>0.999
Largest HCC nodule, n (%)				0.35	0.12, 0.58	0.005
≤2 cm	188 (36%)	168 (38%)	20 (22%)			
>2 cm	341 (64%)	272 (62%)	69 (78%)			
Tumor number, n (%)				0.06	−0.16, 0.29	0.579
Single	358 (68%)	300 (68%)	58 (65%)			
Multiple	171 (32%)	140 (32%)	32 (35%)			
Era of HCC diagnosis, n (%)				0.39	0.16, 0.62	0.014
2000–2004	36 (6.8%)	30 (6.8%)	6 (6.7%)			
2005–2009	103 (19%)	74 (17%)	29 (33%)			
2010–2014	145 (27%)	123 (28%)	22 (25%)			
2015–2019	143 (27%)	126 (29%)	17 (19%)			
2020–2024	102 (19%)	87 (20%)	15 (17%)			
Number of AFP measurements (median, IQR)	7 (4, 10)	6 (4, 10)	7 (4, 10)	−0.06	−0.29, 0.17	0.753
Interval between AFP measurements, days (median, IQR)	164 (107, 197)	161 (108, 192)	175 (105, 234)	−0.28	−0.51, −0.05	0.124
Follow-up duration after HCC diagnosis, years (median, IQR)	2.5 (0.9, 5.3)	2.7 (1.0, 5.4)	1.3 (0.4, 4.4)	0.14	−0.09, 0.36	0.003

AFP, alpha-fetoprotein; CI, Confidence Interval; HCC, hepatocellular carcinoma; HBV, hepatitis B virus; HCV, hepatitis C virus; SMD, Standardized Mean Difference. *p*-values were obtained from the Wilcoxon rank-sum test for continuous variables and from Pearson’s chi-squared or Fisher’s exact test for categorical variables.

**Table 2:** Fit statistics for two- to five-class latent class mixed models of log-AFP trajectories using natural cubic splines (df = 3) for time.

Number of Classes	Log-Likelihood	Parameters	AIC	BIC	Entropy	% in Classes
1	−4960.573	8	9937.147	9971.315	1.000	100.0
**2**	**−4460.943**	**13**	**8947.885**	**9003.408**	**0.879**	**83.2/16.8**
3	−4346.445	18	8728.891	8805.768	0.881	17.4/4.0/78.6
4	−4286.655	23	8619.309	8717.542	0.892	2.3/4.0/17.0/76.7
5	−4265.222	28	8586.444	8706.032	0.862	2.8/4.0/16.8/72.8/3.6

AIC, Akaike Information Criterion; BIC, Bayesian Information Criterion. The two-class model (in bold) was selected as the primary model on the basis of parsimony, entropy and clinical interpretability.

**Table 3:** Univariable and Multivariable Cox proportional-hazards model of overall survival.

Variable	HR	95% CI	*p*-Value	Adjusted HR	95% CI	*p*-Value
Age (per 10-year increase)	1.07	0.97–1.18	0.20	1.07	0.96–1.18	0.232
Sex (male vs. female)	1.01	0.81–1.25	>0.90	0.95	0.76–1.19	0.679
AFP trajectory class (Rising vs. Non-rising)	1.33	1.02–1.73	0.033	1.01	0.77–1.33	0.920
Cirrhosis (yes vs. no)	3.97	1.27–12.4	0.017	5.25	1.67–16.45	0.004
HCC size (per 1 cm increase)	1.19	1.15–1.24	<0.001	1.17	1.12–1.22	<0.001
BCLC stage A (vs. stage 0)	1.50	1.17–1.94	0.002	1.14	0.86–1.50	0.356
BCLC stage B–D (vs. stage 0)	3.07	2.33–4.04	<0.001	2.11	1.56–2.86	<0.001

HR, hazard ratio; CI, confidence interval; AFP, alpha-fetoprotein; BCLC, Barcelona Clinic Liver Cancer.

## Data Availability

Data described in the manuscript and analytic code will be made available upon request.

## Supplementary Materials

The following supporting information can be downloaded at: https://www.mdpi.com/article/10.3390/cancers18182900/s1, Supplementary Figure S1: Flow of patients through the study; Supplementary Table S1: Comparison of characteristics between patients included and excluded from the longitudinal AFP trajectory analysis; Supplementary Table S2: Collinearity Diagnostics (GVIF); Supplementary Table S3: Multivariable Cox Regression: Tumor > 2 cm Subgroup (n = 302); Supplementary Table S4: Multivariable Cox Regression: Tumor ≤ 2 cm Subgroup (n = 227).

## Abbreviations

AFP	Alpha-fetoprotein
AASLD	American Association for the Study of Liver Diseases
AIC	Akaike Information Criterion
ALD	Alcohol-related liver disease
BCLC	Barcelona Clinic Liver Cancer
BIC	Bayesian Information Criterion
BMI	Body mass index
CI	Confidence interval
CT	Computed tomography
EASL	European Association for the Study of the Liver
GALAD	Gender, Age, AFP-L3, AFP, and DCP score
GLP-1	Glucagon-like peptide-1
HBV	Hepatitis B virus
HCC	Hepatocellular carcinoma
HCV	Hepatitis C virus
HIS	Health Information System
HR	Hazard ratio
IQR	Interquartile range
LCMM	Latent class mixed model
LOESS	Locally estimated scatterplot smoothing
MASLD	Metabolic dysfunction-associated steatotic liver disease
MRI	Magnetic resonance imaging
PIVKA-II	Protein induced by vitamin K absence or antagonist-II
SGLT2	Sodium-glucose cotransporter 2
