# Supplementary material for: Distinct Alpha-Fetoprotein Trajectories Preceding Hepatocellular Carcinoma Diagnosis: A Latent Class Mixed-Model Analysis
Source: Cancers (Basel). 2026 Sep 8;18(18):2900. doi: 10.3390/cancers18182900 (PMC13604369; doi:10.3390/cancers18182900)
Supplement: Supplementary file 1 [file cancers-18-02900-s001.zip › cancers-4462206-supplementary.pdf]

Supplementary Material

Supplementary Figure S1. Flow of patients through the study.

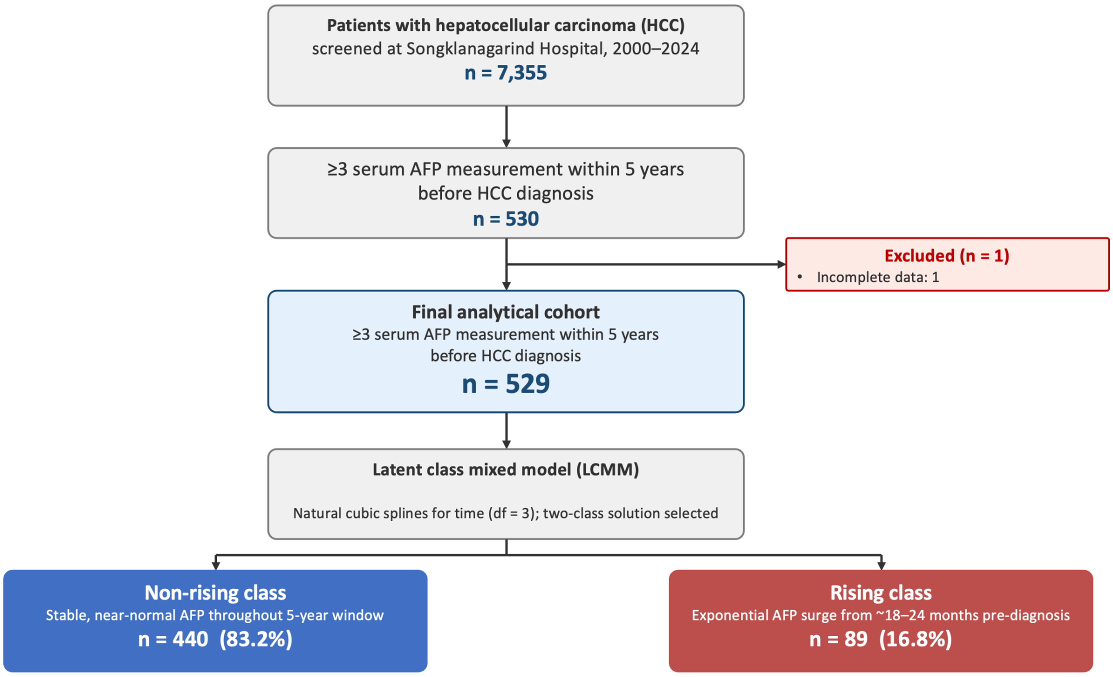

A total of 7,355 patients with hepatocellular carcinoma (HCC) registered at Songklanagarind Hospital between 2000 and 2024 were screened. Among them, 530 patients had at least three serum AFP measurements within the five years before HCC diagnosis and met the inclusion criteria. One patient was excluded because of incomplete data, leaving 529 patients in the final analytical cohort. Latent class mixed modelling (LCMM) with natural cubic splines for time (df = 3) identified two distinct AFP trajectory classes: a non-rising class (n = 440, 83.2%) characterised by relatively stable AFP levels throughout the five-year pre-diagnostic period, and a rising class (n = 89, 16.8%) characterised by a marked exponential increase in AFP beginning approximately 18–24 months before HCC diagnosis.

Supplementary Table S1. Comparison of characteristics between patients included and excluded from the longitudinal AFP trajectory analysis.

**Characteristic**	**Overall N = 7,355**	**Excluded N = 6,826**	**Included N = 529**	**p-value**
**Male sex, n (%)**	5,633 (77%)	5,257 (77%)	376 (66%)	<0.001
**Female sex, n (%)**	1,722 (23%)	1,531 (23%)	191 (34%)	
**Age, years**	60 (52, 69)	60 (51, 68)	63 (56, 70)	<0.001
**Number of AFP measurements within 5 years**	0 (0, 1)	0 (0, 1)	7 (4, 10)	<0.001
**AFP, ng/mL**	66 (9, 2,990)	246 (13, 8,919)	11 (6, 34)	<0.001
**Hemoglobin**	11.87 (10.40, 13.30)	11.70 (10.29, 13.12)	12.41 (11.19, 13.72)	<0.001
**White blood cell count**	7.6 (5.6, 10.5)	8.0 (5.9, 10.9)	5.9 (4.6, 7.4)	<0.001
**Platelet count**	177 (114, 258)	190 (123, 275)	113 (79, 160)	<0.001
**Blood urea nitrogen**	14 (11, 19)	14 (11, 19)	13 (11, 17)	<0.001
**Creatinine**	0.93 (0.77, 1.12)	0.93 (0.77, 1.13)	0.93 (0.78, 1.09)	0.500
**Total bilirubin**	1.2 (0.7, 2.3)	1.3 (0.7, 2.5)	1.0 (0.7, 1.7)	<0.001
**Direct bilirubin**	0.61 (0.32, 1.35)	0.64 (0.33, 1.50)	0.47 (0.27, 0.84)	<0.001
**Alanine aminotransferase (ALT)**	49 (32, 78)	51 (32, 82)	40 (28, 56)	<0.001
**Aspartate aminotransferase (AST)**	85 (53, 153)	93 (57, 172)	55 (40, 77)	<0.001
**Alkaline phosphatase**	160 (105, 269)	178 (114, 293)	107 (81, 141)	<0.001
**Total protein**	7.50 (7.08, 7.95)	7.50 (7.00, 8.00)	7.52 (7.22, 7.89)	0.042
**Albumin**	3.50 (3.01, 4.00)	3.50 (3.00, 3.90)	3.84 (3.33, 4.24)	<0.001

Supplementary Table S2. Collinearity Diagnostics (GVIF).

Variable	GVIF	Df	GVIF^(1/(2×Df))
**Age**	1.065	1	1.032
**Sex**	1.081	1	1.039
**AFP trajectory class**	1.039	1	1.019
**Cirrhosis**	1.010	1	1.005
**HCC size**	1.432	1	1.196
**BCLC stage**	1.450	2	1.097

GVIF^(1/(2×Df)) < 2.5 is the conventional threshold for acceptable collinearity. All values are well within acceptable limits, confirming that multicollinearity is not a confound in the multivariable model.

Supplementary Table S3. Multivariable Cox Regression: Tumor > 2 cm Subgroup (n = 302).

**Variable**	**N**	**Adjusted HR**	**95% CI**	**p-value**
**Age (per 10-year increment)**	302	1.05	0.93–1.19	0.4
**Sex (male vs. female)**	302	0.84	0.64–1.10	0.2
**AFP trajectory class (Rising vs. Non-rising)**	302	1.27	0.93–1.73	0.14
**HCC size (per 1 cm increase)**	302	1.18	1.13–1.24	< 0.001
**HCC stage**	302			
BCLC-0-A (reference)		—	—	
Other stage (B/C/D)		2.22	1.71–2.89	< 0.001

Abbreviations: HR, hazard ratio; CI, confidence interval; AFP, alpha-fetoprotein; HCC, hepatocellular carcinoma; BCLC, Barcelona Clinic Liver Cancer.

Supplementary Table S4. Multivariable Cox Regression: Tumor ≤ 2 cm Subgroup (n = 227).

Variable	N	Adjusted HR	95% CI	p-value
**Age (per 10-year increment)**	227	1.11	0.93–1.32	0.3
**Sex (male vs. female)**	227	1.22	0.86–1.73	0.3
**AFP trajectory class (Rising vs. Non-rising)**	227	1.11	0.66–1.88	0.7
**HCC size (per 1 cm increase)**	227	0.98	0.59–1.62	> 0.9
**HCC stage**	227			
BCLC-0 (reference)		—	—	
BCLC-A		1.59	1.08–2.33	0.019
Other stage (B/C/D)		1.65	0.90–3.04	0.11

Abbreviations: HR, hazard ratio; CI, confidence interval; AFP, alpha-fetoprotein; HCC, hepatocellular carcinoma; BCLC, Barcelona Clinic Liver Cancer.
